# The lysosomotrope GPN mobilises Ca^2+^ from acidic organelles

**DOI:** 10.1242/jcs.256578

**Published:** 2021-03-11

**Authors:** Yu Yuan, Bethan S. Kilpatrick, Susanne Gerndt, Franz Bracher, Christian Grimm, Anthony H. Schapira, Sandip Patel

**Affiliations:** 1 Department of Cell and Developmental Biology, UCL, London WC1E 6BT, UK; 2 Department of Pharmacy – Center for Drug Research, Ludwig-Maximilians University, Munich 81377, Germany; 3 Walther Straub Institute of Pharmacology and Toxicology, Faculty of Medicine, Ludwig-Maximilians University, Munich 80336, Germany; 4 Department of Clinical Neurosciences, UCL Institute of Neurology, London NW3 2PF, UK

**Keywords:** Lysosomes, Ca^2+^, NAADP, Two-pore channels

## Abstract

Lysosomes are acidic Ca^2+^ stores often mobilised in conjunction with endoplasmic reticulum (ER) Ca^2+^ stores. Glycyl-L-phenylalanine 2-naphthylamide (GPN) is a widely used lysosomotropic agent that evokes cytosolic Ca^2+^ signals in many cells. However, whether these signals are the result of a primary action on lysosomes is unclear in light of recent evidence showing that GPN mediates direct ER Ca^2+^ release through changes in cytosolic pH. Here, we show that GPN evoked rapid increases in cytosolic pH but slower Ca^2+^ signals. NH_4_Cl evoked comparable changes in pH but failed to affect Ca^2+^. The V-type ATPase inhibitor, bafilomycin A1, increased lysosomal pH over a period of hours. Acute treatment modestly affected lysosomal pH and potentiated Ca^2+^ signals evoked by GPN. In contrast, chronic treatment led to more profound changes in luminal pH and selectively inhibited GPN action. GPN blocked Ca^2+^ responses evoked by the novel nicotinic acid adenine dinucleotide phosphate-like agonist, TPC2-A1-N. Therefore, GPN-evoked Ca^2+^ signals were better correlated with associated pH changes in the lysosome compared to the cytosol, and were coupled to lysosomal Ca^2+^ release. We conclude that Ca^2+^ signals evoked by GPN most likely derive from acidic organelles.

## Abstract (precis)

**Summary:** Methods of releasing calcium from lysosomes are limited but characterization of the effects of GPN in primary cultured human fibroblasts confirmed that it probably targets acidic organelles.

## INTRODUCTION

Release of stored Ca^2+^ is a ubiquitous means to generate cytosolic Ca^2+^ signals (Clapham, 2007). The endoplasmic reticulum (ER) forms a large Ca^2+^ store housing well-characterised Ca^2+^ channels, buffers and pumps (Bootman and Bultynck, 2019; Clapham, 2007). In addition, a number of acidic organelles also serve as readily releasable Ca^2+^ stores (Morgan et al., 2011; Patel and Docampo, 2010; Patel and Muallem, 2011). Chief among the so-called acidic Ca^2+^ stores are lysosomes that maintain a luminal pH of ∼4.5 and a Ca^2+^ concentration of ∼500 µM, similar to the ER (Christensen et al., 2002). These stores are mobilised through activation of Ca^2+^-permeable channels, such as two-pore channels (TPCs) and transient receptor potential mucolipins (TRPMLs), by signalling molecules, such as nicotinic acid adenine dinucleotide phosphate (NAADP) and phosphatidylinositol 3,5-bisphosphate [PI(3,5)P_2_] (Grimm et al., 2012; Patel, 2015). Owing to their small size, lysosomes generate local Ca^2+^ signals. However, these signals are often amplified by the ER to generate global Ca^2+^ elevations (Kilpatrick et al., 2016b; Morgan et al., 2013; Patel et al., 2001; Penny et al., 2015). Reciprocally, ER-derived Ca^2+^ signals can be tempered by Ca^2+^ uptake into the lysosome (Lopez-Sanjurjo et al., 2013). Such bidirectional inter-organellar communication is thought to occur at membrane contact sites between lysosomes and the ER (Kilpatrick et al., 2013; Penny et al., 2014). Local and global Ca^2+^ signalling through lysosomes and late endosomes regulates numerous processes, ranging from organelle morphology (Kilpatrick et al., 2017; Lin-Moshier et al., 2014) and trafficking of various cargoes, including viruses (Sakurai et al., 2015) through to exocytosis (Brailoiu et al., 2003; Davis et al., 2012; Samie et al., 2013) and functional potential in immune cells (Goodridge et al., 2019). Importantly, deregulated lysosomal Ca^2+^ signalling is associated with diseases such as lysosomal storage disorders (Fares and Greenwald, 2001; Kilpatrick et al., 2016a; Lloyd-Evans et al., 2008), underscoring the biomedical need to delineate lysosomal Ca^2+^ signalling in full.

Studying acidic Ca^2+^ stores in live cells is challenging. Lysosomal Ca^2+^ is difficult to measure directly due to the extreme pH and proteolytic environment. Additionally, there are few cell-permeable activators of lysosomal Ca^2+^ release channels available. In this context, the modified dipeptide glycyl-L-phenylalanine 2-naphthylamide (GPN) has long been used to probe lysosomal Ca^2+^ content (Haller et al., 1996). This compound is a substrate for the thiol protease cathepsin C (CTSC, also known as dipeptidyl peptidase 1) and readily permeates cells (Jadot et al., 1984). Consequently, its cleavage inside cathepsin C^+^ compartments, such as lysosomes, is thought to cause osmotic changes resulting in rupture. The leak of lysosomal contents, such as Ca^2+^, can be readily measured in the cytosol, thus providing an indirect measure of lysosomal Ca^2+^ content. GPN evokes Ca^2+^ signals in numerous cell types and selectively inhibits Ca^2+^ signals by activation of TPCs and TRPMLs (Calcraft et al., 2009; Dong et al., 2010; Kilpatrick et al., 2016b).

The mechanism underpinning lysosomal Ca^2+^ uptake is unclear. Much indirect evidence points to Ca^2+^-H^+^ exchange whereby the steep proton gradient across the lysosomal membrane is used to drive antiport of Ca^2+^ into the lysosome (Morgan et al., 2011; Patel and Docampo, 2010; Patel and Muallem, 2011). Collapsing the H^+^ gradient with inhibitors of the V-type ATPase, such as bafilomycin A1, reduces lysosomal Ca^2+^ levels (Christensen et al., 2002). Like GPN, such treatment abrogates Ca^2+^ responses evoked directly by NAADP and by select extracellular Ca^2+^ mobilising agonists that couple to NAADP production (Churchill et al., 2002; Yamasaki et al., 2004). Conversely, bafilomycin A1 reportedly potentiates Ca^2+^ responses evoked by IP_3_-forming agonists (similar to GPN), IP_3_ and thapsigargin (Lopez-Sanjurjo et al., 2013), presumably as a result of impaired Ca^2+^ uptake by lysosomes. Ca^2+^-H^+^ exchangers (CAX) have been molecularly identified and characterised in plants, microorganisms (such as yeast) and more recently in select animals (Melchionda et al., 2016). However, they are absent in the genomes of humans and other placental mammals (Melchionda et al., 2016). Alternative mechanisms to fill lysosomes with Ca^2+^ include coupled Na^+^/Ca^2+^ and Na^+^/H^+^ exchange or H^+^-independent Ca^2+^ uptake, possibly by P-type ATPases, such as ATP13A2 (Garrity et al., 2016; Narayanaswamy et al., 2019; Patel and Docampo, 2010).

A recent study has challenged the mechanism by which GPN evokes changes in cytosolic Ca^2+^ (Atakpa et al., 2019). Work by Atakpa et al. (2019) pointed out that GPN is a weak base and alkalizes the cytosol, and that it is this change in cytosolic pH that underpins the Ca^2+^ signals. Moreover, the authors provided evidence that GPN-evoked Ca^2+^ signals are independent of cathepsin C and suggest that they derive directly from the ER and not the lysosome (Atakpa et al., 2019). These provocative findings impact many previous studies using GPN (Morgan et al., 2020). We therefore characterised the effects of GPN in fibroblasts, which mount robust Ca^2+^ responses (Kilpatrick et al., 2013). We confirm an effect of GPN on cytosolic pH but dissociate this from changes in Ca^2+^. We further show that GPN-evoked Ca^2+^ signals are inhibited by a chronic elevation in lysosomal pH. Our data support an action of GPN at the lysosome.

## RESULTS

### GPN and NH_4_Cl differentially affect cytoplasmic Ca^2+^ and pH

**Fig. 1.**
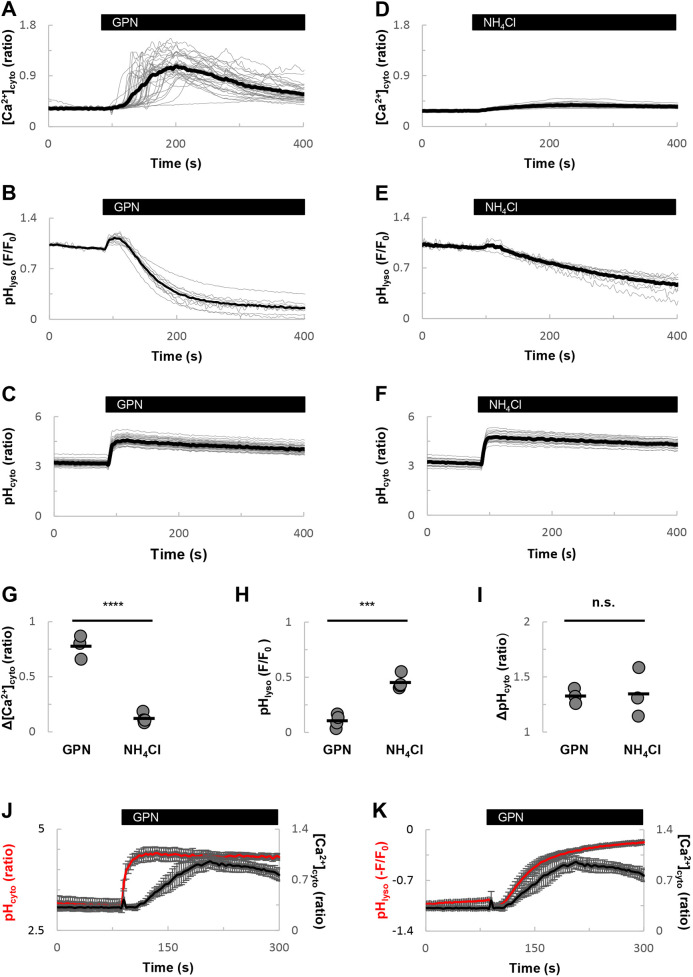
**GPN and NH_4_Cl differentially affect cytoplasmic Ca^2+^ and pH.** (A-C) Effects of GPN (200 µM) on cytosolic Ca^2+^ (A), lysosomal pH (B) and cytosolic pH (C) in fibroblasts. Time courses were obtained from cells loaded with Fura-2, LysoTracker Red and BCECF, respectively. Data are expressed as the indicated fluorescence ratio (for Ca^2+^ or cytosolic pH) or fluorescence (F) relative to initial fluorescence (F_0_) for LysoTracker Red. Grey traces represent recordings for all individual cells from a typical field of view. Black traces represent the population average. An increase in Fura-2 and BCECF ratios corresponds to an increase in cytosolic Ca^2+^ and pH, respectively. A decrease in LysoTracker Red fluorescence corresponds to an increase in lysosomal pH. (D-F) Similar to A-C, except cells were stimulated with NH_4_Cl (5 mM). (G-I) Summary data quantifying effects of GPN and NH_4_Cl on cytosolic Ca^2+^ (G), lysosomal pH (H) and cytosolic pH (I). Each point represents the average of all labelled cells in a field of view from an independent experiment (*n*=3-4). ***P<0.001; ****P<0.0001; n.s., not statistically significant (independent-samples *t*-tests). (J,K) Comparison of the effects of GPN on Ca^2+^ relative to changes in cytosolic (J) and lysosomal (K) pH. Data are mean±s.e.m. from 3-4 independent experiments.

To explore the mechanism underlying GPN-evoked Ca^2+^ signals, we investigated the relationship between changes in Ca^2+^ and pH in primary cultured human fibroblasts. As shown in Fig. 1A, GPN evoked robust cytosolic Ca^2+^ signals in Fura-2 loaded cells with a characteristic delay. This effect was associated with time-dependent decreases in fluorescence of the fluorescent acidotrope LysoTracker Red (Fig. 1B), as reported previously (Kilpatrick et al., 2013).

To examine the effect of GPN on cytosolic pH, we used the ratiometric indicator BCECF. As shown in Fig. 1C, GPN also evoked an increase in cytoplasmic pH consistent with recent findings (Atakpa et al., 2019). Summary data quantifying the changes in Ca^2+^ and pH are presented in Fig. 1G-I. Comparison of the kinetics of the various responses shows that the effect of GPN on cytoplasmic pH was rapid, peaking ∼1 min before the peak of the Ca^2+^ response (Fig. 1J). In contrast, the increase in lysosomal pH mirrored the increase in cytosolic Ca^2+^ (Fig. 1K).

To further probe the relationship between Ca^2+^ and pH, we examined the effects of the alkalizing agent, NH_4_Cl. NH_4_Cl had little effect on cytosolic Ca^2+^ levels (Fig. 1D) but increased both lysosomal (Fig. 1E) and cytosolic (Fig. 1F) pH. The increase in cytosolic pH was comparable to that evoked by GPN (Fig. 1I).

Taken together, these data dissociate changes in cytoplasmic pH from Ca^2+^, both kinetically and pharmacologically.

### V-type ATPase inhibition progressively increases lysosomal pH

Bafilomycin A1 is often used to disrupt Ca^2+^ homoeostasis by acidic organelles by preventing H^+^-dependent Ca^2+^-uptake (Christensen et al., 2002; Churchill et al., 2002). However, as reported recently, bafilomycin A1 potentiates GPN-evoked Ca^2+^ signals (Atakpa et al., 2019). To determine the effect of bafilomycin A1 on GPN-evoked Ca^2+^ signals in fibroblasts, we first characterised its effect on lysosomal pH.

**Fig. 2.**
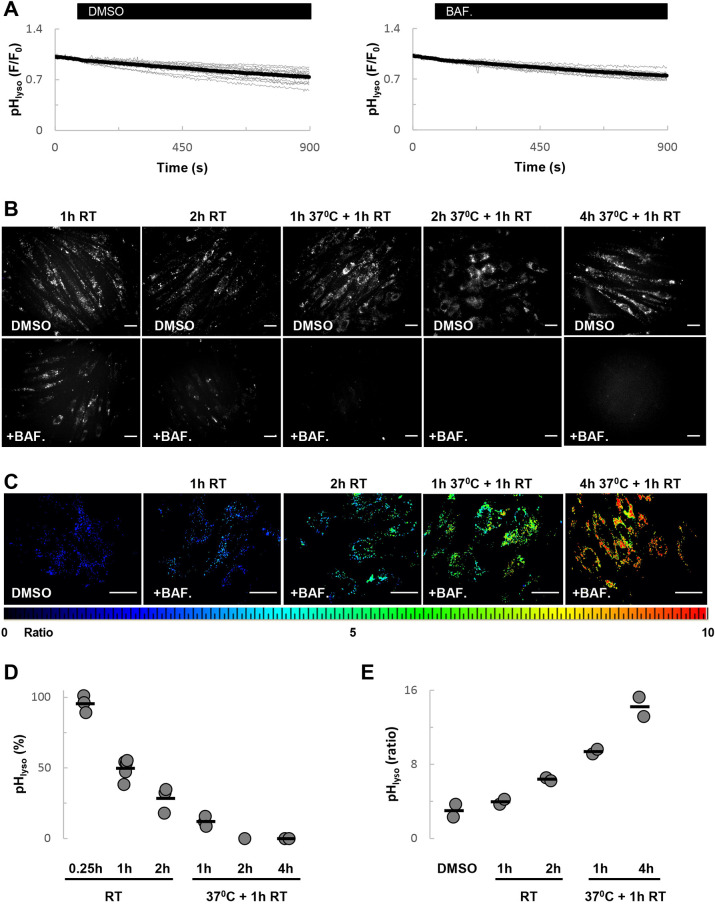
**V-type ATPase inhibition progressively increases lysosomal pH.** (A) Effects of DMSO [0.1% (v/v)] and bafilomycin A1 (1 µM) on the fluorescence of LysoTracker Red. (B-C) Representative images showing LysoTracker Red fluorescence (B) or fluorescein-dextran ratio (C) of cells treated with bafilomycin A1 (1 µM) or DMSO for the indicated time. Cells were maintained at room temperature or in culture conditions. In the latter, the final hour of incubation was performed at room temperature in HBS. Scale bars: 50 µm. An increase in fluorescein dextran ratio corresponds to an increase in lysosomal pH. (D,E) Summary data (*n*=1-5) quantifying the effect of bafilomycin A1 on LysoTracker Red fluorescence (D) and fluorescein-dextran ratio (E). The data in D are expressed relative to DMSO.

As shown in Fig. 2A, time-lapse imaging of cells at room temperature upon acute treatment with bafilomycin A1 up to ∼15 min had little effect on LysoTracker Red fluorescence. Preincubation of cells with bafilomycin A1 for 1 h, similar to the conditions reported in Atakpa et al. (2019), before labelling, reduced fluorescence by ∼50% (Fig. 2B). Longer preincubations (2 h) largely, but not completely, eliminated fluorescence (Fig. 2B).

To define conditions that result in complete collapse of the pH gradient, we performed additional experiments in which cells were treated with bafilomycin A1 for up to 5 h. To maintain cell viability, cells were incubated with bafilomycin A1 for 1 to 4 h under culture conditions. As shown in Fig. 2B, LysoTracker Red fluorescence was eliminated under these conditions. The effects of bafilomycin A1 on LysoTracker Red fluorescence data are quantified in Fig. 2D.

In a second approach, we measured lysosomal pH ratiometrically using cells labelled with fluorescein dextran. As shown in Fig. 2C, and quantified in Fig. 2E, the fluorescence ratio increased in a progressive manner in the presence of bafilomycin A1 similar to loss of LysoTracker Red fluorescence. In sum, these data show that inhibition of the V-type ATPase is associated with progressive alkalisation of the lysosome.

### Acute V-type ATPase inhibition potentiates Ca^2+^ signals evoked by GPN and thapsigargin

**Fig. 3.**
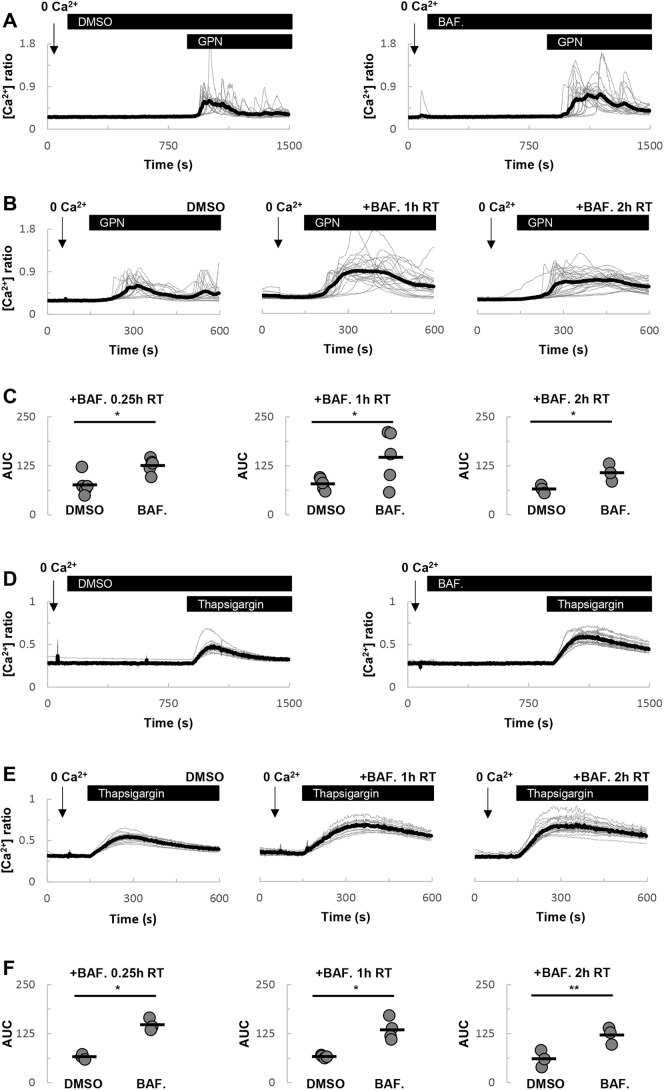
**Acute V-type ATPase inhibition potentiates Ca^2+^ signals evoked by GPN and thapsigargin.** (A) Effects of acute DMSO [0.1% (v/v)] and bafilomycin A1 (1 µM) treatment on GPN-evoked Ca^2+^ signals. Experiments were performed at room temperature in the absence of external Ca^2+^. (B) Effects of DMSO or a 1-2 h treatment with bafilomycin A1 (1 µM) on GPN-evoked Ca^2+^ signals. (C) Summary data quantifying the effect of bafilomycin A1 on the area under the curve (AUC) of the GPN responses (*n*=3-5). (D-F) Similar to A-C, except cells were stimulated with thapsigargin (1 µM) in place of GPN (*n*=3-4). *P<0.05, **P<0.01 (paired-samples *t*-tests).

Having defined the effect of bafilomycin A1 on lysosomal pH, we proceeded to examine its effect on GPN-evoked Ca^2+^ signals. As shown in Fig. 3A, GPN-evoked Ca^2+^ signals were potentiated by a short (∼15 min) incubation with bafilomycin A1. This potentiation is consistent with recent findings (Atakpa et al., 2019) but occurred under conditions in which lysosomal pH was not demonstrably altered (Fig. 2). Similar potentiation was observed following longer incubations for 1 or 2 h at room temperature (Fig. 3B). The effects of bafilomycin A1 on GPN-evoked Ca^2+^ signals are quantified in Fig. 3C.

In parallel experiments, we examined the effects of bafilomycin A1 on ER-derived Ca^2+^ signals. Here, we used thapsigargin to probe ER Ca^2+^ content. Again, as reported previously (Lopez-Sanjurjo et al., 2013), thapsigargin-evoked Ca^2+^ signals were enhanced by bafilomycin A1, whether added acutely (Fig. 3D) or following preincubation for up to 2 h (Fig. 3E). But again, this effect appeared to be independent of the time of bafilomycin A1 treatment (Fig. 3F). Thus, bafilomycin A1 potentiates both GPN and thapsigargin-evoked Ca^2+^ signals in a manner that is apparently independent of lysosomal pH changes.

### Sustained V-type ATPase inhibition selectively inhibits GPN-evoked Ca^2+^ signals

**Fig. 4.**
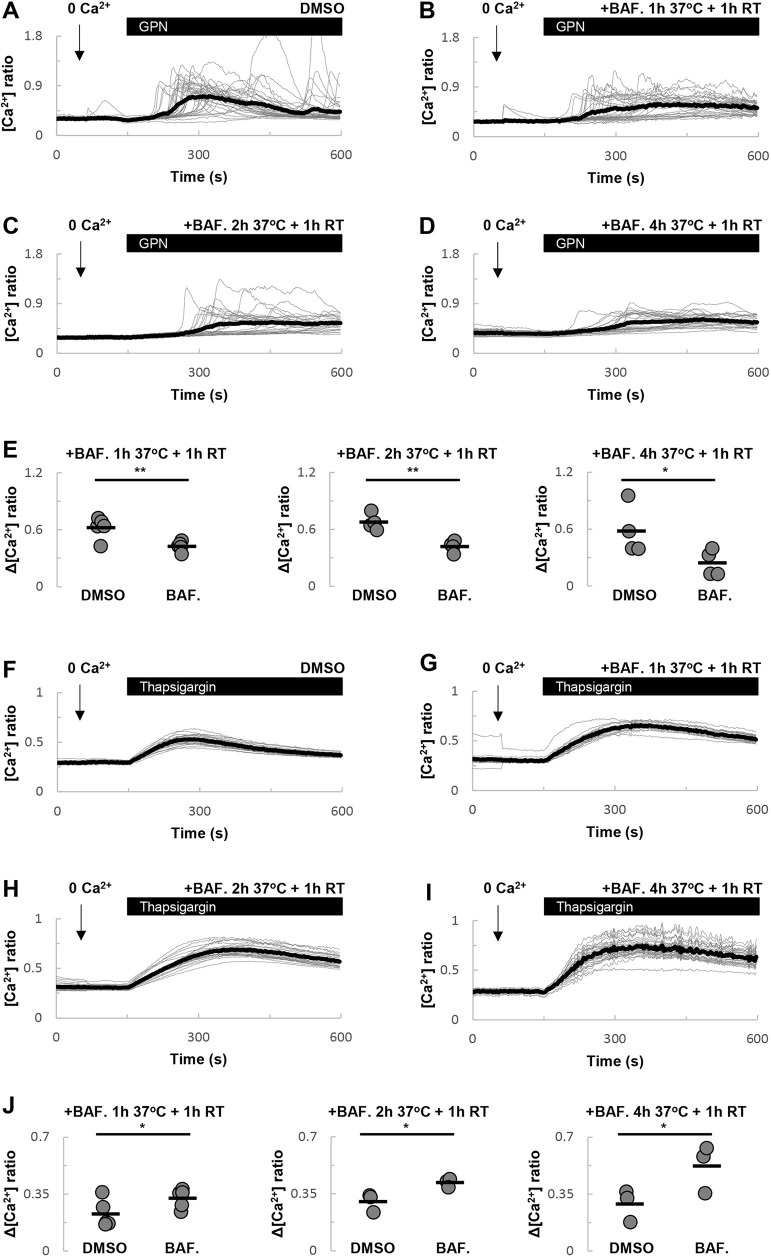
**Sustained V-type ATPase inhibition selectively inhibits GPN-evoked Ca^2+^ signals.** (A-D) Effects of DMSO (A), or a 2 h (B), 3 h (C) or 5 h (D) treatment with bafilomycin A1 (1 µM) on GPN-evoked Ca^2+^ signals. Cells were incubated under culture conditions except for the final 1 h when they were loaded with Fura-2 at room temperature in the continued presence of bafilomycin A1. (E) Summary data quantifying the effect of bafilomycin A1 on the magnitude of the GPN responses (*n*=4-5). (F-J) Similar to A-E, except cells were stimulated with thapsigargin (1 µM) in place of GPN (*n*=3-5). *P<0.05, **P<0.01 (paired-samples *t*-tests).

To further investigate the effect of lysosomal pH on GPN-evoked Ca^2+^ signals, we examined the effects of chronic bafilomycin A1 treatment, which has a more profound alkalizing effect on the lysosome (Fig. 2). As shown in Fig. 4A-D, incubation for up to 5 h with bafilomycin A1 inhibited GPN-evoked Ca^2+^ signals. These data, quantified in Fig. 4E, show a twofold decrease in Ca^2+^ signals in the presence of bafilomycin A1.

We also examined the effects of chronic bafilomycin A1 treatment on thapsigargin-evoked Ca^2+^ signals. Similar to the shorter treatments (Fig. 3), bafilomycin A1 potentiated thapsigargin responses (Fig. 4F-J).

Thus, prolonged alkalisation of lysosomes selectively inhibits the effects of GPN.

### GPN blocks TPC2-dependent Ca^2+^ signals

**Fig. 5.**
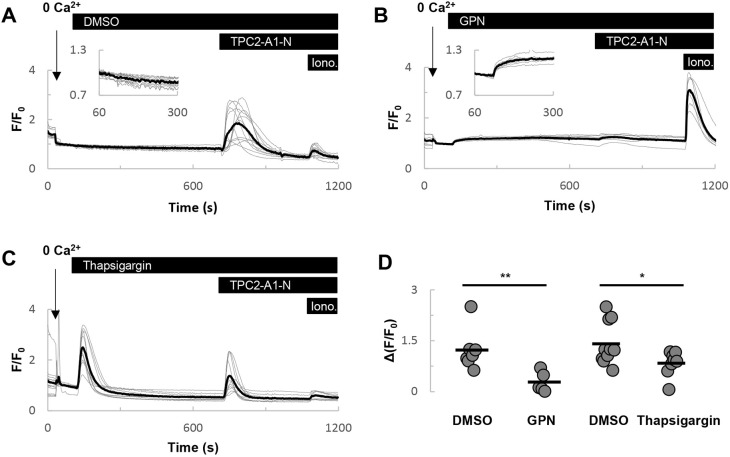
**GPN inhibits TPC2-dependent Ca^2+^ signals.** (A-C) Effects of acute challenge with DMSO (A), 200 µM GPN (B) or 1 µM thapsigargin (C) on Ca^2+^ signals evoked by TPC2-A1-N (30 µM). Experiments were performed using HeLa cells expressing TPC2-GCaMP6s in the absence of external Ca^2+^. Ionomycin (2 µM) was added at the end of the experiments. An increase in GCaMP6s fluorescence corresponds to an increase in cytosolic Ca^2+^. (D) Summary data quantifying the effect of GPN and thapsigargin on the magnitude of the TPC2-A1-N-evoked responses (*n*=5-10). *P<0.05, **P<0.01 (Mann–Whitney U-tests).

Finally, we examined the effects of GPN on Ca^2+^ signals evoked by activation of TPC2. Here we took advantage of the recently described TPC2 agonist TPC2-A1-N, which mimics the actions of NAADP (Gerndt et al., 2020). In HeLa cells expressing TPC2 fused to the genetically encoded Ca^2+^ indicator GCaMP6s, TPC2-A1-N (30 µM) evoked robust Ca^2+^ signals in the absence of extracellular Ca^2+^ (Fig. 5A), as reported previously (Gerndt et al., 2020). Treatment of cells with GPN (200 µM) also evoked a Ca^2+^ signal, but the responses were modest. TPC2-A1-N failed to affect cytosolic Ca^2+^ levels upon prior challenge with GPN (Fig. 5B).

For comparison, we examined the effects of depleting ER Ca^2+^ stores with thapsigargin. Thapsigargin-evoked Ca^2+^ signals that were larger than those evoked by GPN (Fig. 5B,C). Thapsigargin treatment partially blocked the effects of TPC2-A1-N (Fig. 5C). Collectively, these data summarised in Fig. 5D show that Ca^2+^ signals evoked by activation of a lysosomal ion channel are preferentially inhibited by GPN.

## DISCUSSION

GPN is a lysosomotropic agent used widely for Ca^2+^ signalling studies and beyond. However, its traditional mechanism of action has been recently challenged. Here, we combined measurements of Ca^2+^ and pH in the cytosol together with measurements of lysosomal pH to investigate how GPN evokes Ca^2+^ signals in fibroblasts. Our data support the canonical view whereby GPN targets acidic organelles to mediate Ca^2+^ signals.

As a cathepsin C substrate, GPN has been used for decades to disrupt lysosomes through lysosome membrane permeabilization (Jadot et al., 1984). Consequently, the concomitant increases of lysosomal pH and cytosolic Ca^2+^ that GPN demonstrably evokes have naturally been ascribed to H^+^ and Ca^2+^ release from the lysosome. However, the recent findings that the ionic changes induced by GPN are independent of cathepsin C and lysosome permeabilization demands scrutiny of the underlying mechanism (Atakpa et al., 2019). Our data confirm that GPN is a weak base and that similar to NH_4_Cl, increases the pH of the cytosol and lysosomes (as well as, presumably, other cellular compartments) (Fig. 1). But, two lines of presented evidence argue against the proposal that it is the increase in cytosolic pH which drives the Ca^2+^ changes. The first relates to kinetics. GPN evokes a rapid pH change but a slower Ca^2+^ response in the cytosol (Fig. 1J). The second is based on the differential effects of NH_4_Cl. NH_4_Cl increases cytosolic and lysosomal pH as expected, but not Ca^2+^ (Fig. 1D-F). NH_4_Cl mediated increases in Ca^2+^ in some cells (Danthuluri et al., 1990) but not others (Fasolato et al., 1991; Yagodin et al., 1999) pointing to cell-type-specific differences.

Consistent with an action of GPN on acidic organelles are data presented here showing that bafilomycin A1 inhibits GPN-evoked Ca^2+^ signals (Fig. 4). These data concur with previous studies in other cell types (Gunaratne et al., 2018; Zhang et al., 2014). However, this inhibition in fibroblasts required extended incubation periods with bafilomycin A1. Ca^2+^ uptake into lysosomes is thought to be dependent on the pH gradient. Accordingly, collapse of the pH gradient with lysosomotropic agents would be expected to prevent Ca^2+^ uptake. However, for store depletion to occur in response to bafilomycin A1, both H^+^ and Ca^2+^ must leak out of the lysosome. In fibroblasts, H^+^ leak appears to be slow because a 1 h incubation with bafilomycin A1 at room temperature, a not uncommon condition, only reduced LysoTracker Red fluorescence by ∼50% (Fig. 2). The mechanism underlying Ca^2+^ leak from the lysosome (and the more extensively studied ER for that matter) is unknown. If leak of Ca^2+^ from the lysosome is also slow then this might explain why only prolonged bafilomycin A1 treatment inhibits GPN-evoked Ca^2+^ signals. It might also explain why neither bafilomycin A1 nor NH_4_Cl acutely induce a Ca^2+^ signal. Interestingly, incubation of cells with bafilomycin A1 for 2 h only modestly increased lysosomal pH when the incubation included a 1 h culture period at 37°C relative to cells that were maintained at room temperature throughout. Yet, these treatments had reciprocal effects on GPN-evoked Ca^2+^ signal such that inhibition was only noted in the former conditions. This implies that the effects of bafilomycin A1 are temperature-dependent, perhaps pointing to accelerated lysosomal Ca^2+^ leak at elevated temperature. Of relevance here is the original study identifying lysosomal-like Ca^2+^ stores as NAADP targets (Churchill et al., 2002). Bafilomycin A1 readily inhibited Ca^2+^ uptake into vesicular preparations enriched in lysosome markers. NAADP-induced Ca^2+^ release was also blocked by bafilomycin A1 in intact cells, but only upon a second challenge with NAADP. These data were interpreted as the target Ca^2+^ stores being non-leaky to Ca^2+^ requiring the prior opening of channels to effect Ca^2+^ depletion. Human fibroblasts and sea urchin eggs appear similar with respect to lysosomal Ca^2+^ handling and its sensitivity to bafilomycin A1.

The effect of acute treatment with bafilomycin A1 on Ca^2+^ signals is notable in two respects. First, it shows that GPN-evoked Ca^2+^ signals were potentiated. This confirms recent findings (Atakpa et al., 2019), which were interpreted as arguing against an action of GPN on acidic organelles. Second, it shows that thapsigargin-evoked Ca^2+^ signals are similarly potentiated. Again, these findings are not inconsistent with previous work (Lopez-Sanjurjo et al., 2013). Such potentiation was interpreted previously as an inhibitory effect of bafilomycin A1 on lysosomal Ca^2+^ uptake, possibly through disruption of contact between lysosomes and the ER (Atakpa et al., 2018), thereby preventing tempering of ER-derived Ca^2+^ signals by lysosomes. But in fibroblasts, the potentiation of both GPN- and thapsigargin-evoked Ca^2+^ signals by bafilomycin A1 (Figs 3,4) appeared to be independent of V-type ATPase inhibition as it did not correlate with the slow changes in lysosomal pH (Fig. 2). Perhaps most striking was the effect of short treatment with bafilomycin A1, which had little effect on LysoTracker Red staining but potentiated the Ca^2+^ responses to both GPN (Fig. 3A) and thapsigargin (Fig. 3D). The mechanism underlying this effect is unclear at present but clearly worthy of future attention.

Previous studies demonstrated a block of NAADP- but not IP_3_/cADPR-mediated Ca^2+^ signals by GPN and bafilomycin-A1 (Churchill et al., 2002; Yamasaki et al., 2004). Such a block is consistent with a large body of evidence indicating an action of NAADP on acidic organelles. The selective nature of the block is difficult to reconcile with a sole action of GPN on the ER. So too are more contemporary findings in fibroblasts from Parkinson's disease patients (Kilpatrick et al., 2016a) and mast cells from TPC1 knockout mice (Arlt et al., 2020), demonstrating reduced GPN-evoked Ca^2+^ signals in the face of enhanced ER-derived Ca^2+^ responses. To further critique the action of GPN, we took advantage of the recent identification of small-molecule cell-permeable TPC2 agonists that mimic the actions of NAADP and PI(3,5)P_2_ (Gerndt et al. 2020). Notably, the Ca^2+^ mobilizing activity of the NAADP-like agonist (TPC2-A1-N) was abolished by prior treatment of cells with GPN (Fig. 5). This is similar to a block of TRPML1-mediated Ca^2+^ signals evoked by the TRPML agonist, ML-SA1 (Kilpatrick et al., 2016b). In contrast, thapsigargin only partially inhibited TPC2-A1-N action. This dual sensitivity is entirely consistent with the ‘trigger’ hypothesis whereby NAADP-mediated Ca^2+^ signals derive from acidic organelles and are amplified by the ER. The more pronounced block by GPN relative to thapsigargin is again inconsistent with GPN targeting ER Ca^2+^ stores exclusively. This set of experiments was performed in HeLa cells, and in our hands the Ca^2+^ response to acute GPN challenge was modest relative to fibroblasts, which may reflect cell-type-specific differences.

In sum, we conclude that in fibroblasts, GPN most likely evokes Ca^2+^ release by targeting bafilomycin A1-sensitive lysosomal Ca^2+^ stores. Exactly how GPN releases Ca^2+^ from lysosomes requires further work given that GPN-evoked Ca^2+^ signals are reportedly independent of cathepsin C and amplification by IP_3_ receptors (Atakpa et al., 2019). Nevertheless, our data affirm the role of acidic organelles in mediating Ca^2+^ signals in response to lysosomotropic compounds.

## MATERIALS AND METHODS

### Cell culture

Primary cultured human dermal fibroblasts or HeLa cells were maintained in Dulbecco's modified Eagle medium supplemented with 10% (v/v) fetal bovine serum, 100 units/ml penicillin and 100 µg/ml streptomycin (all from Invitrogen) at 37°C in a humidified atmosphere with 5% CO_2_. Cells were passaged by scraping (fibroblasts) or following trypsin treatment (HeLa cells), and plated onto coverslips for imaging. For experiments with HeLa cells, coverslips were coated with poly-L-lysine. Cells were mycoplasma-negative.

### Measurement of cytosolic Ca^2+^ and pH

Cytosolic Ca^2+^ and pH were measured independently using the fluorescent ratiometric indicators Fura-2 and BCECF, respectively. All experiments were performed in HEPES-buffered saline (HBS) comprising 1.25 mM KH_2_PO_4_, 2 mM CaCl_2_, 2 mM MgSO_4_, 3 mM KCl, 156 mM NaCl, 10 mM glucose and 10 mM HEPES (pH 7.4; all from Sigma-Aldrich). For measurement of Ca^2+^, cells were incubated with Fura-2 AM (2.5 µM) and 0.005% (v/v) pluronic acid (from Invitrogen) for 1 h in HBS. Fura-2 was excited at 340/380 nm and emitted fluorescence was captured using a 440 nm long pass filter and a 20× objective. For the measurement of pH, cells were incubated with BCECF-AM (5 µM) and 0.005% v/v pluronic acid (Invitrogen) for 30 min in HBS. BCECF was excited at 490/440 nm and emitted fluorescence was captured using a 515 nm long pass filter and a 20× objective.

### Measurement of lysosomal pH

Lysosomal pH was measured using the fluorescent acidotrope LysoTracker Red and the ratiometric fluorescent indicator fluorescein. Cells were labelled with LysoTracker Red (100 nM, Invitrogen) for 15 min at room temperature in HBS. The dye was excited at 568 nm and emitted fluorescence was captured using a 590 nm filter and a 20× objective. This was used as a proxy for lysosomal pH given that LysoTracker Red is a hydrophobic weak base that accumulates in acidic compartments in a pH-dependent manner (DiCiccio and Steinberg, 2011). To measure lysosomal pH more directly, cells were loaded with dextran-conjugated fluorescein (1 mg/ml; MW 10,000; from Invitrogen) by endocytosis for ∼6 h in culture. Cells were subsequently chased overnight in dextran-free culture medium to label lysosomes. Fluorescein was excited at 488/425 nm and emitted fluorescence was captured using a 515 nm long pass filter and a 40× objective. Fluorescence of this compound is directly dependent on protonation providing a more quantitative readout of lysosomal pH.

### Measurement of TPC2 activity

TPC2 activity was measured using TPC2-GCaMP6s as described by Gerndt et al. (2020). Briefly, HeLa cells expressing GCaMP6s fused to the cytosolic C-terminus of TPC2 were stimulated with the TPC2 agonist TPC2-A1-N (Gerndt et al., 2020). GCaMP6s was excited at 470 nm and emitted fluorescence was captured using a 515 nm long pass filter and a 40× objective.

### Epifluorescence microscopy

After dye labelling or transfection, cells were washed with HBS and mounted in a 1 ml imaging chamber (Biosciences Tools) prior to microscopy. Epifluorescence images were captured every 3 s with a cooled coupled device camera (TILL photonics) attached to an Olympus IX71 inverted fluorescence microscope fitted with a monochromator light source. Cells were stimulated with 200 µM GPN (Santa Cruz Biotechnology), 1 µM thapsigargin (Santa Cruz Biotechnology) or 30 µM TPC2-A1-N (synthesized as described by Gerndt et al., 2020) at room temperature. In some experiments, Ca^2+^ was omitted from the HBS.

### Bafilomycin A1 treatment

Cells were acutely treated with 1 µM bafilomycin A1 (Cell Signaling Technology) during recording, or preincubated for up to 2 h at room temperature. For extended bafilomycin A1 treatments, cells were incubated for up to 4 h in culture followed by an additional 1 h at room temperature in HBS in the continued presence of bafilomycin A1. For Fura-2 and LysoTracker Red measurement, the dyes were loaded in the presence of bafilomycin A1 at room temperature. For fluorescein-dextran measurements, bafilomycin A1 was added after the chase period.

### Data analysis

Fluorescence signals were quantified on an individual cell basis and averaged for all cells in a given field of view (up to 42 cells). For Fura-2 and BCECF, the maximal fluorescence ratio increase was calculated by subtracting the basal ratio from the peak ratio obtained in response to a given stimulus. In some Fura-2 experiments, the area under the curve was calculated by integrating the fluorescence ratios following subtraction of the basal ratio. For LysoTracker Red and TPC2-GCaMP6s recordings, signals were quantified as the maximal fractional fluorescence change relative to the basal intensity (F/F_0_) upon stimulation. Basal ratios and intensities were averaged for 60 to 90 s of recording or for shorter periods if there were spontaneous fluctuations, but this was rare. For fluorescein-dextran, the steady-state fluorescence ratio was presented. Data are collated as individual means from independent experiments or as mean±s.e.m. Statistical analyses were performed using Prism 9. Independent-samples *t*-tests, paired-samples *t*-tests or Mann–Whitney U-tests were applied. *P*<0.05 was considered statistically significant.
